# Study of the Effect of an Oral Formulation of Fig and Olive on Rheumatoid Arthritis (RA) Remission Indicators: A Randomized Clinical Trial

**Published:** 2016

**Authors:** Shahnaz Bahadori, Jamshid Salamzadeh, Mohammad Kamalinejad, Mohammad Reza Shams Ardekani, Mansoor Keshavarz, Arman Ahmadzadeh

**Affiliations:** a *Faculty of Traditional Medicine, Tehran University of Medical Sciences, Tehran, Iran. *; b *Food Safety Research Center, Shahid Beheshti University of Medical Sciences, Tehran, Iran.*; c *Department of Clinical Pharmacy, School of Pharmacy, Shahid Beheshti University of Medical Sciences, Tehran, Iran. *; d *Department of Pharmacognosy, School of pharmacy, Shahid Beheshti University of Medical Sciences, Tehran, Iran. *; e *Faculty of Pharmacy, Tehran University of Medical Sciences, Tehran, Iran. *; f *Department of Physiology, School of Medicine, Tehran University of Medical Sciences, Tehran, Iran. *; g *Department of Rheumatology, Loghman-e Hakim Hospital, Shahid Beheshti University of Medical Sciences, Tehran, Iran.*

**Keywords:** Rheumatoid arthritis, Complementary medicine, Fig, Olive, DAS28_ESR

## Abstract

This study was designed to explore the complementary effects of a combination formulation of olive oil, olive and fig fruits on RA remission indicators. A randomized controlled clinical trial was designed. Adult RA patients were randomly divided into two groups receiving routine Disease-modifying antirheumatic drugs (DMARDs) regimen (control group) and routine DMARDs regimen plus the herbal supplementary formulation of olive oil, fig and olive fruits (intervention group). Patients were followed every 4 weeks for total study period of 16 weeks. In addition to demographic and medical history of the patients, the Disease Activity Score with 28-joint counts based on Erythrocyte Sedimentation Rate (DAS28_ESR) were recorded. SPSS (version 22.0) software was used to analyze data, assuming p<0.05 as significance level. 56 patients (control = 27 and intervention = 29), with mean ± sd age of 50.91 ± 12.26 years completed the study. Repeated measures analysis revealed that differences between remission indicators in the two study groups were not statistically significant, however, there was a p = 0.03 for the within-subjects contrast test of the Patient Global Assessment (PtGA), approving a nonlinear change for PtGA with respect to time. No between groups differences in adjunct drug therapy pattern for disease flares were seen. In conclusion, although, non-significant changes in the study variable of DAS28_ESR is in agreement with few previous reports, nevertheless, trends in its reduction in the intervention group along with the significant delayed PtGA score improvements occurred in the intervention group convince us to suggest further investigations on the supplementary olive and fig products, with a longer follow up periods.

## Introduction

Rheumatoid arthritis (RA) is a common, chronic, inflammatory, autoimmune disease with indefinite etiology affecting about 0.5-1.0% of the world population (1, 2). According to the reports from Middle East countries the prevalence of RA is 0.14% to 0.55% (3). As stated by a report, prevalence of RA in Iran is around 0.32% (4). The current data support a role for genetic and environmental factors in disease risk (1). Immune reactions resulted from the contribution of genetic and environmental factors, lead to development of synovitis, joint damage, structural bone damage which eventually cause swelling, pain, morning stiffness, fatigue, disability, as well as emotional, social, and economic challenges for the patient and health systems (2). In addition, the corresponding systemic inflammation may result in extraarticular comorbidities and disorders involving multiple organ systems (5). 

Unfortunately, there is currently no definite cure or means to prevent RA. The available guidelines recommend the prompt diagnosis and well-monitored treatment with Disease Modifying Anti-Rheumatic Drugs (DMARDs) and biologic agents to limit the degree of permanent joint damage (6). Despite advances in diagnosis of RA, existing drug therapies have limited effectiveness and should be used cautiously due to their frequently reported adverse and toxic effects (7). In addition, newly introduced expensive biologic agents are not easily accessible, consequently, patients do not fully benefit from them (8). Therefore, introducing more effective, safer and economically justified alternative treatments for RA have always been a target for current researches.

In this regard, some researchers have focused on traditional herbal and complementary medicines. In addition, there are growing global popularity in using traditional medicines (9). *Rao et al. *in a study investigating rheumatologic patients› perspective on use of complementary and alternative medicines has concluded that these medicines are widely used by patients with rheumatologic conditions (10). Also, Soeken *et al.* in a systematic review conducted between 1966 and 2001 revealed that there is growing interest to use herbal medicines for painful chronic conditions, in particular RA, and recommended to conduct more investigations to clarify the safety, efficacy and potential drug interactions of the herbal preparations (7). 

Olive (*Olea Europe*) and fig (*Ficus carica*) are amongst those herbal medicines that are frequently studied and documented to have beneficial anti-inflammatory, immunomodulatory, antimicrobial, anticancer, chemopreventive, analgesic and anti-oxidant effects. They mainly owe their biologic properties to oleic acid and phenolic components (11-27). 

Furthermore, lupeol, a dietary triterpene found in olive and fig fruit, has shown anti-inflammatory, anti-arthritic, anti-mutagenic and anti-malarial activity (12, 28). In addition, based on ethnopharmacological documents and folkloric believes, olive and fig have been used in inflammatory disorders such as inflammatory swellings and hard swellings (22).

There are few experimental studies and clinical trials that have worked on the efficacy of different parts of the *Olea Europe* (olive) and *Ficus carica* (fig) plants in inflammatory joint disorders in particular RA. In a clinical trial, Berbert *et al.* have shown that combination of fish oil and olive oil has more dominant remission effect on RA symptoms compared to that of fish oil alone (21). Similarly, in a study comparing effects of the evening primrose oil and olive oil on RA patients, olive oil could significantly reduce pain and articular index on the patients at 6 months (29). An *ex-vivo* study by Park *et al.* showed that hexane soluble fraction of the common fig is a potent inhibitor of osteoclastogenesis and therefore may have therapeutic effects on bone-destructive processes such as osteoporosis, rheumatoid arthritis, and periodontal bone resorption (30). An animal study on the effect of methanolic extract of *Ficus carica* Linn. Leaves on a rat model of RA, also revealed that the extract has anti-inflammatory effects and could ameliorate cell influx and exudation to the site of the inflammatory response (31). No human study on the effect of fig, alone or in combination with olive, on RA was found. The American College of Rheumatology (ACR) in a position statement concerning complementary and alternative medicine (CAM) for rheumatic diseases, issued in 2012, has cited that “The ACR supports the integration of those modalities proven to be safe and effective by scientifically rigorous clinical trials published in the biomedical peer review literature.”(32).

Given that the ingestion of *Olea Europe* and *Ficus carica* plants could ameliorate the signs and symptoms of RA, along with long-standing experience of Iranian traditional medicine in herbal and nutritional therapy, we decided to design the current study with the aim of exploring the complementary effects of a combination formulation of olive oil, olive and fig fruits on RA remission indicators.

**Table 1:** Demographic data and baseline medical and medication history of the study groups

**Variable**	**Subgroup**	**Intervention group**	**Control group**	**p value**
Age (year)	N/A	50.38 ± 12.25^+^	51.48 ± 12.49	0.74
Sex	Male	5 (17.24%)	3 (11.11%)	0.71
	Female	24 (82.76%)	24 (88.88%)	
RA history (years)	N/A	6.69 ± 4.44 [6.00, 4.00-8.50]*	7.09 ± 5.20 [6.00, 3.00-12.00]	0.98
Main referral reason	New case	3 (10.34%)	4 (14.81%)	0.81
	Flare-up	13 (44.83%)	10 (37.04%)	
	Follow up	13 (44.83%)	13 (48.15%)	
Concurrent rheumatoid disease	Yes	9 (31.03%)	9 (33.33%)	0.85
	No	20 (68.97%)	18 (66.67%)	
Comorbidity	Yes	8 (27.59%)	10 (37.04%)	0.45
	No	21 (72.41%)	17 (62.96%)	
Concurrent drug	Yes	8 (27.59%)	7 (25.93%)	0.89
	No	21 (72.41%)	20 (74.07)	
No of tender joints	N/A	[3.00, 2.00-6.50]	[3.00, 1.00-4.00]	0.24
No of swollen joints	N/A	[3.00, 2.00-5.00]	[2.00, 1.00-4.00]	0.07
Patient global assessment	N/A	81.38 ± 19.95 [80.00, 70.00-100.00]	79.26 ± 23.85 [80.00, 50.00-100.00]	0.98
ESR	N/A	26.41 ± 19.78 [21.00, 12.00-33.50]	21.63 ± 17.38 [15.00, 10.00-32.00]	0.23
DAS28_ESR	N/A	4.81 ± 1.00	4.31 ± 1.05	0.07
Menopause female patients	Yes	16 (66.67%)	15 (62.50%)	0.76
	No	8 (33.33%)	9 (37.50%)	

N/A: Not Applicable; RA: Rheumatoid Arthritis; **+** Continuous data are presented as mean ± standard deviation;

* [Median, Interquartile range] for discrete or non-normally distributed variables; ESR: Erythrocyte Sedimentation Rate.

**Figure 1:**
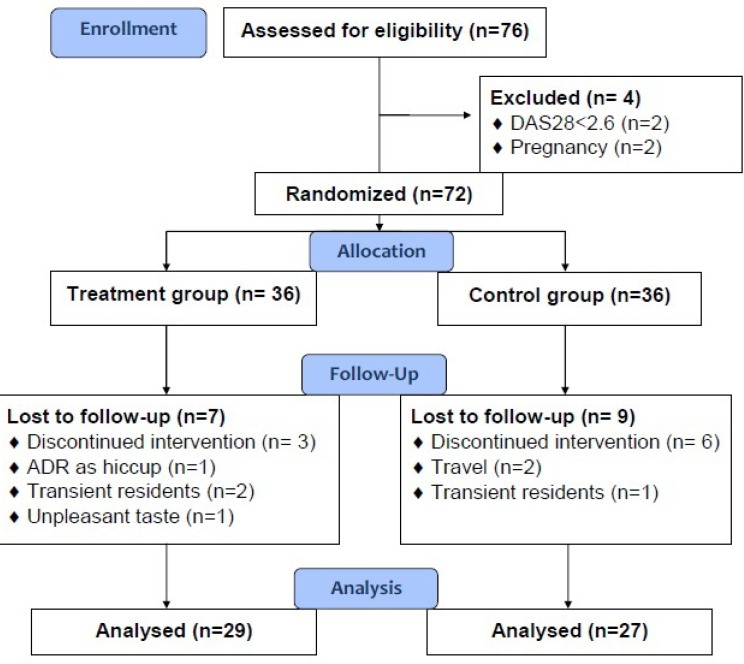
Study flowchart

**Table2:** Results of the repeated measures ANOVA

**Remission indicator**	**Repeated Measures ANOVA Result**	
	**Multivariate p value**	**Within-subjects contrasts p value**
DAS28_ESR	0.55	0.33
No of Tender joints	0.60	0.84
No of swollen joints	0.68	0.81
ESR	0.43	0.29
PtGA	0.25	0.03

ANOVA: Analysis of Variance; DAS: Disease Activity Score with 28-joint counts; ESR: Erythrocyte Sedimentation Rate; PtGA: Patient Global Assessment of disease activity

**Table 3:** Results of the comparison between reduction (baseline - week 16) in the measures of RA remission in the control and intervention groups

**Remission indicator**	**Intervention group**	**Control group**	**p value**
DAS28_ESR	1.76 ± 1.39^+^	1.37 ± 1.35	0.34
No of Tender joints	[2.00, 1.00-4.00]*	[2.00, 0.00-3.00]	0.49
No of swollen joints	[2.00, 1.00-3.25]	[1, 0.00-3.00]	0.45
ESR	7.65 ± 16.16	3.57 ± 11.90	0.33
PtGA	44.09 ± 30.03	25.22 ± 26.26	0.03

DAS: Disease Activity Score with 28-joint counts; ESR: Erythrocyte Sedimentation Rate; PtGA: Patient Global Assessment of disease activity; + Continuous data are presented as mean ± standard deviation;

* [Median, Interquartile range] for discrete or non-normally distributed variables.

**Figure 2:**
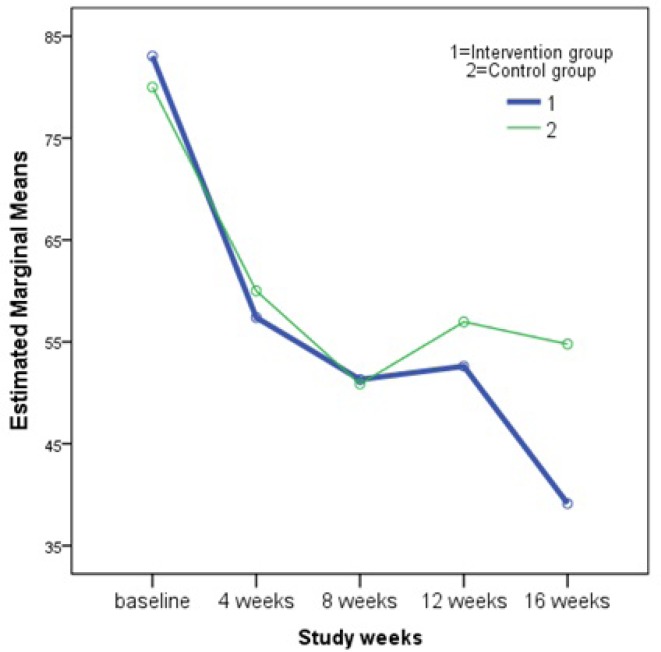
Changes in the mean PtGA index during the study weeks

## Experimental

*Material and Methods*

A randomized controlled parallel group clinical trial of routine DMARDs regimen *vs.* routine DMARDs regimen plus an oral supplementary formulation of olive oil, olive and fig fruits (as add on therapy) was designed. Routine DMARDs regimen included methotrexate, hydroxychloroquine, azathioprine, sulfasalazine. The study was approved by the ethics committee of the Tehran University of Medical Sciences and was registered at the Iranian Registry of Clinical Trials with registration ID of IRCT2013122015876N1.

*Study population and sample size estimation*

Patients with definite diagnosis of RA referring to the in- and out-patient rheumatology departments of the Loghman-e Hakim University Hospital, Tehran, Iran, were randomly divided into two study groups receiving routine DMARDs regimen (control group) and routine DMARDs regimen plus the herbal supplementary formulation (an edible semisolid mixture) of fig and olive (intervention group). Sampling was carried out during September 2014 to August 2015. Estimated sample size for each group was 27 patients, with α = 0.05 and power = 80%. Patients were followed for 16 weeks. For each patient, an in-person follow up visit was arranged every 4 weeks. Therefore, there were 5 repeated measurements of the study variables for each individual patient; one at the time of enrollment (baseline) and 4 during the 4-week intervals. 

*Study herbal supplement*

The herbal supplement used in this trial was a combination of olive oil, olive fruit and fig fruit with proportional amounts of 2:5:1 w/w formulated as a semisolid mixture. An appropriate stabilizer, i.e. ascorbic acid, was also added to protect the product from oxidation. Ensuring fresh formulation intake by the patients and for checking their compliance, study formulation was prepared and delivered to the patients in regular 10-day intervals. Patients were trained to take 15 grams (equal to 1 table-spoonful) of the mixture, t.d.s with meals and were asked not to change the usual dietary intake. They were also taught to keep the herbal medicine in a cool place away from heat and light. 

Stability testing of the herbal supplement was performed by 3 times measurements, done in 10 days intervals. For this purpose, acid value (AV) and peroxide value (PV) were determined using titrimetry methods according to ISO 660:1996 and ISO 3960:2001, respectively, recommended by the Codex Alimentarius Commission (33). No significant changes in the AV (11.82 ± 1.88 mg/Kg) and PV (1.05 ± 0.2 meq/Kg) of the herbal supplement occurred during 20 days of storage at room temperature.

Olive and olive oil used in this study were prepared from the olive trees (*Olea europaea *L.) cultivated at Rudbar city located at the Gilan province in the North of Iran. Fig fruits were purchased as dried form, originated from common fig trees (*Ficus carica* L.) in Estahban city at the Fars province. Voucher specimens of the olive and fig fruits were preserved in the Herbarium of the School of Pharmacy, Shahid Beheshti University of Medical Sciences. (Herbarium No: 1115 and 8105, respectively).

*Study outcomes*

Primary outcome measure determined in this research, was Disease Activity Score with 28-joint counts based on Erythrocyte Sedimentation Rate (DAS28_ESR) recommended by the American College of Rheumatology as a measure of RA disease activity that is an applied criteria for clinical practice (34). It is also often considered as a “gold standard” by which RA disease activity is measured (35). Four components of the DAS28_ESR including tender joint count, swollen joint count, erythrocyte sedimentation rate and patient global assessment of disease activity (PtGA) were also determined at baseline and in each of the 4 follow up visits. PtGA measures the overall way RA affects the patient at a point in time. It includes a statement such as “Considering all of the ways your arthritis has affected you, how do you feel your arthritis is today?” It can be scored from 0 (very well) to 100 (very poor) (35).

In addition, demographic characteristics of the patients and their medical and medication history were recorded. Any adjuncts therapy for disease flares *i.e.* use of systemic and intraarticular glucocorticoids and non-steroidal anti-inflammatory drugs (NSAIDs) were listed. Furthermore, possible side effects contributed to the herbal supplement were observed and documented.

*Inclusion and exclusion criteria*

All RA patients, male or female, over 18 years old with a DAS28_ESR score > 2.6 that were under treatment with DMARDs entered the study. Exclusion criteria included biologic agent therapy in the last 6 months, patients with concurrent rheumatoid diseases and gout, patients with uncontrolled diabetes, regular consumption of olive and/or fig in the last 3 months, history of intraarticular corticosteroid in the last 3 months and pregnancy. In addition, exit criteria were any major change in the usual dietary intake, using other complementary and alternative medicine during the study period, any addiction to psychotropic agents and opioids, any severe adverse effect or intolerance to drug therapy including the herbal supplement, refusal for inclusion in the study and poor or noncompliant patients. Noncompliance was defined as consumption of less than 80% or more than 120% of the prescribed dose of investigational product. Patients were asked to sign a written informed consent form before they enroll in trial.

*Statistical analysis*

Comparison of the demographic and baseline medical and medication history of the patients in two study groups were done using the Student’s t-test and Mann-Whitney U-test for quantitative data, and the Chi-square and Fisher’s exact tests for qualitative data. A repeated-measures analysis of variance (ANOVA) was applied to test any differences in repeated measurements of the primary outcomes between control and intervention groups. p values < 0.05 were considered as significance level.

## Results

Overall, from 76 patients who were assessed for eligibility to enter the study, 72 patients fulfilled the criteria, of them 56 patients, 27 in the control group and 29 in the intervention group, completed the study. Study flowchart is illustrated in Figure 1. Demographic data and baseline medical and medication history of the study groups and results of their comparison between two groups are presented at the Table 1. As it is seen, no statistically significant difference existed in the demographic features, as well as medical and medication history of the patients in the control and intervention groups. Compliance of the patients in the intervention group was 93.88 ± 7.06 percent, ranging from 80% to 100%, confirming their appropriate adherence to the dosage regimen of the herbal supplement.

Results of the repeated measures ANOVA on the RA remission indicators are shown in Table 2. Also, results of the comparison between reduction (baseline - week 16) in the measures of RA remission in the control and intervention groups are presented in the Table 3.

As it is perceived in the Table 2, differences between remission indicators in the two groups of control and intervention were not statistically significant. In other words, our intervention supplement could not improve the remission indicators of RA, however, there was a p = 0.03 for the within-subjects contrast test of the patient global assessment of disease activity (PtGA). This means that there was a significant difference between PtGA of the control group and that of intervention group which was not changing in a linear pattern with respect to time. As it is illustrated in the Figure 2, changes in the PtGA in the intervention group is comparable with that of control group at first, however we can see how the two groups diverged at the 12^th^ week and this continues to a wider difference at the end of study (week 16). To illuminate this effect, a student’s t-test was performed to compare differences between baseline and final PtGA scores in two study groups of control (25.22 ± 26.26) and intervention (44.09 ± 30.03). The test result confirmed significant effect (p = 0.03) of the herbal supplement. Then, the level of global health and well-being of the patients in the intervention group was higher than that of the patients in the control group.

Regarding the number of patients for whom intraarticular glucocorticoid was administered during the study period, comparison of the control (2 out of 27) and intervention (7 out of 29) groups revealed no significant difference. Likewise, total cumulative dose of steroids was not different between control (5.05 ± 1.59 mg) and intervention (5.56 ± 1.98 mg) groups. Alike glucocorticoids, number of patients receiving NSAIDs was not significantly different between control (1 out of 27) and intervention (4 out of 29) groups through the 16 weeks of study. 

No significant adverse reaction contributed to the investigational herbal supplement was seen in the study groups except one case with severe unclassified hiccup in the intervention group that leaded to his withdrawal from the study (Figure 1.).

## Discussion

Phytochemical exploration of the different parts of olive and fig trees are in agreement with the results of *in-vitro* and *in-vivo* studies confirming anti-inflammatory, analgesic and anti-oxidant effects of herbal supplements obtained from these plants (11-29, 31). On the other hand, ethnopharmacological experiences approve their indication in relevant debilitating diseases (7, 22). To re-examine those conventional beneficial effects of olive and fig, we decided to conduct the current study on RA, which is pathophysiologically related to primary inflammatory and autoimmune processes (2).

Overall, 56 patients completed our study of which 48 (85.71%) patients were female. Mean ± sd age of the study patients was 50.91 ± 12.26 years, ranging from 26 to 74 years. Age and sex distribution of the patients in our study are in accordance with the epidemiological studies that show in all countries, prevalence of RA is higher in females compared to males and prevalence increases with age (1-4).

High rate of compliance of the intervention group (93.88 ± 7.06 %) to take the herbal supplement regularly as administered by the researchers, endorses the previous reports that there is growing interest in herbal therapies among persons with RA and other rheumatologic conditions (7, 10). This was noticeably observed in the current study, when all patients in the intervention group were gratified for being included in the study. 

Results of the Repeated Measures ANOVA showed that the herbal supplement (a combination of olive oil, olive and fig fruits) did not have a significant impact on the major rheumatoid arthritis disease activity measures including DAS28_ESR, tender joint count, swollen joint count, erythrocyte sedimentation rate and the PtGA. However, further analysis of the latter measure verified that there is a nonlinear significant relationship between PtGA and the time (Figure 2), which displays that at the end of 16 weeks of follow up, PtGA in the intervention group has reduced greatly compared to that of the control group. Importance of the PtGA index is emphasized by the American College of Rheumatology members who rated the PtGA and the Provider Global Assessment of disease activity (PrGA) as the most feasible tools for use in clinical practice (34). Our findings on PtGA support the assumption that a longer course of supplement usage could lead to greater remission in RA, which can be reflected in the values of the disease activity measures. This assumption is in agreement with the results obtained by Brzeski *et al.* that showed a significant reduction in morning stiffness of RA patients with gamma-linolenic acid (as primrose oil 6 g/day) at 3 months and reduction in pain and articular index at 6 months with olive oil (6 g/day) (29). In another research by Sköldstam *et al*., who studied the impact of 12-week Mediterranean diet on remission index of DAS28, Health Assessment Questionnaire (HAQ) and a health survey of quality of life (Short Form-36 (SF-36)) in RA patients, it was again observed that the difference between treatment groups was notable only after the second half of the trial (30). 

Our results showed that the usage pattern of systemic and intraarticular steroids as well as NSAIDs were unaffected. Sköldstam *et al.* also found similar results on NSAIDs use in RA patients (30). 

In conclusion, although non-significant changes in the study variables of DAS28_ESR, tender joint count, swollen joint count and ESR observed in this study, are in overall agreements with the previous reports revealing that the available evidence does not support current use of complementary and alternative medicine (CAM) in the management of RA (36), nevertheless, their positive trends in the intervention group (table3) along with the significant delayed PtGA score improvements occurred in the intervention group convince us to suggest further investigations on the supplementary olive and fig products, with a longer follow up periods.
